# Supplementary material for: Workers’ Perception Heat Stress: Results from a Pilot Study Conducted in Italy during the COVID-19 Pandemic in 2020
Source: Int J Environ Res Public Health. 2022 Jul 4;19(13):8196. doi: 10.3390/ijerph19138196 (PMC9266050; doi:10.3390/ijerph19138196)
Supplement: Supplementary file 1 [file ijerph-19-08196-s001.zip › ijerph-1756420-supplementary.pdf]

NATIONAL HEAT AND WORK RISK SURVEY - WORKLIMATE PROJECT (BRIC INAIL 2019)

CALL FOR PARTICIPATION IN THE NATIONAL HEAT AND WORK SURVEY

Climate change is causing an increase in the frequency and intensity of heat waves during the summer period, and it is estimated that about 30% of the world's population is currently exposed to health-critical heat conditions for at least 20 days a year. Workers, in particular those who spend most of their activities outdoors, are among those most exposed to the effects of heat and all weather phenomena in general. The situation this year is further aggravated by the COVID-19 emergency which, among other restrictions, makes it necessary in many situations to use proper personal protective equipment and/or hygiene measures such as so-called 'community masks' (which are intended to reduce the circulation of the virus in daily life and are not subject to any particular certification) and which may further contribute to heat stress.

The objective of this national survey is to investigate the perception and knowledge of the effects of heat in the workplace in order to identify intervention strategies to reduce heat risk for the occupational sector.

The survey questionnaire was developed ad hoc within the INAIL 2019 research activities of the BRIC (Bando Ricerca In Collaborazione) WORKLIMATE project by researchers Michela Bonafede, Miriam Levi, Alessandro Marinaccio, Alessandro Messeri, Marco Morabito, Emma Pietrafesa and the entire Worklimate project group.

Completion of the questionnaire, which is anonymous and confidential and takes about 15/20 minutes, does not require correct or incorrect answers, but only answers that reflect your personal experience and your experiences. The completeness of the answers is essential for the validity of the research. Any comments or requests for further information can be sent to the following e-mail addresses: marco.morabito@ibe.cnr.it; m.bonafede@inail.it

Pursuant to the legislation on the protection of personal data (EU Regulation 2016/679-General Data Protection Regulation- GDPR- applicable since 25 May 2018), we inform you that the data you submit will be collected and stored in an appropriate manner, analysed in an anonymous and aggregated form and will be used exclusively for scientific research purposes.

The results of the research in which you participate may be published in aggregate form, so your identity will remain anonymous. Your contribution to this survey is valuable.

We thank you for your cooperation!

By continuing, you confirm your willingness to participate in our research and express your consent to the processing of your data. * (*Mandatory field)

Section A: DEMOGRAPHIC AND SOCIO-OCCUPATIONAL DATA

1. **Gender***

Men

Woman

Other

2. **If you are female, are you pregnant?**

(YES/NO)

3. **Years old* ____________________________**

4. **School degree qualification***

None

Primary school certificate

Junior high school diploma

High school diploma

Bachelor's degree

Master's degree/specialist degree

Postgraduate training (Master's degree, PhD, specialisation, post-doc)

5. Nationality*

Italian

Community

Non-EU

6. During the hot period (between May and September), do you fast for personal reasons (religious/ethical/nutritional...)?*

Response scales: Never, rarely, sometimes, often, always

7 In which geographical area do you work?*

North (Emilia-Romagna, Friuli-Venezia Giulia, Liguria, Lombardia, Piemonte Trentino-Alto Adige, Valle d'Aosta, Veneto)

Centre (Lazio, Marche, Toscana, Umbria)

South and Island (Abruzzo, Basilicata, Calabria, Campania, Molise, Puglia, Sardegna, Sicilia)

8 In which environment do you work?* Choose only one answer option

- Urban-coastal (plain/hill/mountain)
- Urban-hinterland (plain/hill/mountain)
- Rural-coastal (plain/hill/mountain)
- Rural-hinterland (plain/hill/mountain)

9 Marital status*

Single

Married - Accompanied

Separated - Divorced

Widowed

10 If you have sons/daughters, please indicate age and number *

None	One	Two	Three	Four	Over 4
0-2 years					
3-5 years					
6-10 years					
11-13 years					
14-18 years					
Over 18 years					

11 In which sector do you work?*

- Agriculture, forestry and fishing
- Extraction of minerals from quarries and mines
- Manufacturing (e.g. food processing, tobacco industry, textile/timber industry, paper, manufacture of chemicals/pharmaceuticals, rubber and plastic products, metallurgy, manufacture of machinery and equipment, motor vehicle manufacture)
- Electricity, gas, steam and air conditioning supply
- Water supply; sewerage, waste management and remediation activities
- Construction - Building
- Trade
- Transport and storage
- Accommodation and food service activities
- Information and communication services
- Financial and insurance activities
- Real estate activities
- Professional, scientific and technical activities
- Rental, travel agencies, business support services
- Public administration and defence
- Education
- Health and social work
- Artistic, sporting, entertainment and recreational activities

12. What do you do? *

13. For how many years? *

14. Number of employees in your company? *

From 1 to 9 employees

From 10 to 49 employees

From 50 to 249 employees

250 and more employees

15. In what kind of working environment do you currently work?*

Mainly indoor in air-conditioned environment

Mostly indoors in non-air-conditioned environment

Mostly outdoors

Other (specify)

16 What kind of physical activity do you do on average in your workplace?*

From 1 Very light to 5 Very intense

17. Do you work around heat sources such as ovens or welding etc.?*

Yes

Sometimes

No

18 Do you use chemicals in your work? *

Yes

Sometimes

No

19. Does your job require you to wear protective clothing (e.g. overalls, mask, gloves)? *

Yes

Sometimes

No

20 For how many hours during the day do you use COVID-19 masks in your work? (Specify the number of hours) *

None

Other

21. During the hottest time of the year in which months do you work?*

May

June

July

August

September

22.In your professional life do you deal or have you dealt with Occupational Safety and Health (OSH)?*

Yes

No

23. Do you suffer from chronic diseases (multiple answer)? *

No

Diabetes mellitus

Arterial hypertension

Other cardiovascular disease (e.g. heart failure, previous ischemic heart disease, previous stroke, peripheral obliterative arterial disease, arrhythmias) (e.g. heart failure, previous ischaemic heart disease, previous stroke, peripheral obliterative arterial disease, arrhythmias)

Chronic obstructive pulmonary disease

Asthma

Back pain

Rheumatic diseases

Neurological diseases (e.g. epilepsy)

Chronic kidney disease

Other

24. Has a diagnosis been made of infection with the SARS-CoV-2 virus (the coronavirus responsible for COVID-19)? *

Yes

No

Don't know

25. Have you developed COVID-19 disease in a symptomatic form*?

Yes

No

Don't know

Section B: RISK PERCEPTION *

How much do you agree with the following statements? Use this response scale

1. **Strongly disagree**
2. **Disagree**

3. **Neither agree nor disagree**
4. **Agree**
5. **Strongly agree**

26. I feel that my health is threatened by climate change (General perceived risk)

27. I think heat waves endanger my health (General perceived risk)

28. Workers are involuntarily exposed to heat (Voluntary risk)

29. Heat causes an immediate fatal effect for exposed persons (Immediacy effect)

30. Workers exposed to heat have precise knowledge of the risk (Knowledge of the risk)

31. The scientific world has a complete understanding of the heat risk (Knowledge of the risk)

32. The heat risk is a new risk for Italian companies (New)

33. Heat is a potentially lethal risk (Chronic/Catastrophic)

34. Heat is a risk that workers have learned to live with (Common/Terrifying)

35. Heat poses a very low threat to future generations (Future Generations)

36. Preventive measures in the workplace can reduce the severity of the heat risk (Controlling severity)

37. Heat risk damage is observable (Observability)

38. In summer, during my work, I feel exposed to heat (Personal exposure)

39. During a heat wave I feel very much at risk (Personal exposure)

40. During a heat wave there are many workers at risk in Italy (Collective exposure)

41. I am afraid that heat waves will cause me to have an accident at work (Fear of risk)

42. I am afraid that I will get sick because of heat waves (Fear of risk)

43. During a heat wave I am afraid that the risk of transmission of the virus responsible for COVID-19 will increase.

Section C: RISK KNOWLEDGE*

Express how much you agree with the following statements. Use this response scale

1. **Strongly disagree**
2. **Disagree**
3. **Neither agree nor disagree**
4. **Agree**
5. **Strongly agree**

44. Heat can be the cause of accidents for outdoor workers

45. Heat can cause injuries for those working in a non-air-conditioned indoor environment

46. Younger workers are particularly vulnerable during a heat wave

47. Excessive sweating during a heatwave can be a sign of heat stress

48. People with heart disease are at risk of worsening their health during a heat wave

49. Heat-related illnesses can lead to death

50. Dehydration in hot weather predisposes to the development of serious kidney disease

51. There is no evidence that heat waves can cause respiratory diseases

52. People exposed to heat have an increased risk of developing diabetes

53. Heat waves promote the growth of harmful bacteria in water and food

54. Heat waves can be a risk factor for depression and anxiety

55. Due to the shade of buildings, heat waves are less common in cities than in rural areas

56. Heat stress at night is of no importance

57. During a heat wave the risk of transmission of the virus responsible for COVID-19 increases

Section D: ACCIDENTS, PREVENTION MEASURES AND WORK POLICIES

58. **In your work experience, how often would you say that injuries or accidents occur due to (at least partly) hot/high humidity conditions**:

Never

Rarely

Few times

Often

Don't know

WHOEVER ANSWERS "NEVER" AND "DON'T KNOW" MUST MOVE ON TO THE NEXT QUESTION N. 76

For those who answer "Rarely" - "Sometimes" - "Often" continue with the questions

In your opinion, how much did the following work factors/hazards contribute to the occurrence of these heat-related injuries/accidents? (from 1=not at all to 5=fully)

59. Working in a hectic way

60. Wearing personal protective equipment (PPE) that leads to higher body temperatures

61. Working in the sun without access to shade (solar radiation)

62. Working indoors without air conditioner, fan or adequate ventilation

63. Equipment, machinery, tools

64. Electricity

65. Fire, steam, hot surfaces

66. Hazardous environments

In your opinion, how much did the following organisational aspects contribute to the occurrence of these heat-related injuries/accidents? (from 1=not at all to 5=fully)

67. No regulations related to heat or hot environments

68. Regulations not followed

69. Responsibilities not clearly defined

70. No specific health and safety training on heat stress

71. Production targets and timelines

72. Poor supervision

73. Workers are not allowed to take breaks, if necessary

74. Insufficient access to fresh drinking water

75. What types of workers have suffered these heat-related injuries/accidents? MULTIPLE CHOICE

Newly employed workers

Younger workers (up to 29 years old)

Workers between 30-45 years old

Workers aged (46-55)

Workers aged (56-65)

Workers over 65

Workers whose first language is not Italian

Other (specify)__________________________________

76 Was training on the prevention of heat-related injuries carried out in the places where you worked?

Yes

In some companies

No

Don't know

77 What are your main sources of information on the prevention of heat-related diseases in the workplace? MULTIPLE CHOICE

Competent physician

Family doctor

School training

Facebook

Twitter

LinkedIn

Internet

TV and radio

Newspapers

Friends and family

Colleagues

Specific training in my workplace

Flyers or notices in my workplace

None

Other

78 Do you receive warnings or alerts from your employer or manager about the possibility of a heat wave?

No

Yes, with messages

Yes, verbally

Yes, by notices placed at information points

Yes, by company-specific training

Other (Specify)_________________

79 If yes, how satisfied are you with the measures taken in the workplace to reduce the effects of heat?

From 1 Very dissatisfied to 5 Very satisfied

80 In general, how much do you think heat contributes to the loss of productivity?

From 1 not at all to 5 completely

81 To what extent do you think that each of the following conditions can be an obstacle to the prevention of heat-related occupational accidents? (from 1=not an obstacle at all to 5=definitely an obstacle)

1. Lack of awareness among workers that heat can be associated with illness or injury
2. Lack of awareness among workers about the risks of dehydration
3. Lack of awareness by company health and safety managers of the risks from heat
4. Lack of worker training
5. Lack of training of company health and safety officers
6. Lack of commitment by employers to protect health and safety
7. Lack of financial resources
8. Lack of specific guidelines and regulations on hot workplaces risk
9. Lack of compliance with regulations
10. Concerns on the part of employers about loss of productivity
11. Reluctance on the part of employers to allow workers to take breaks as required
12. Attitudes of workers to continue working at all costs
13. Difficulties in assessing heat risks

Thank you for taking part in the survey, if you wish you can leave comments and/or suggestions
